# Major contribution of the RNA-binding domain of NS1 in the pathogenicity and replication potential of an avian H7N1 influenza virus in chickens

**DOI:** 10.1186/s12985-018-0960-4

**Published:** 2018-03-27

**Authors:** Sascha Trapp, Denis Soubieux, Alexandra Lidove, Evelyne Esnault, Adrien Lion, Vanaique Guillory, Alan Wacquiez, Emmanuel Kut, Pascale Quéré, Thibaut Larcher, Mireille Ledevin, Virginie Nadan, Christelle Camus-Bouclainville, Daniel Marc

**Affiliations:** 1 Equipe PIA, UMR1282-ISP Infectiologie et Santé Publique, INRA, 37380 Nouzilly, France; 2 UMR1282 Infectiologie et Santé Publique, Université de Tours, F-37000 Tours, France; 3 INRA UMR 703, APEX, Oniris-La Chantrerie, F-44307 Nantes, France; 4 LUNAM Université, École Nationale Vétérinaire, agro-alimentaire et de l’alimentation Nantes-Atlantique (Oniris), Nantes, France; 5 Centre de Biophysique Moléculaire, CNRS, Orléans, France; 6 IHAP, UMR1225, Université de Toulouse, INRA, ENVT, Toulouse, France

**Keywords:** Influenza A, NS1, Viral replication, Chicken

## Abstract

**Background:**

Non-structural protein NS1 of influenza A viruses harbours several determinants of pathogenicity and host-range. However it is still unclear to what extent each of its two structured domains (i.e. RNA-binding domain, RBD, and effector domain, ED) contribute to its various activities.

**Methods:**

To evaluate the respective contributions of the two domains, we genetically engineered two variants of an H7N1 low pathogenicity avian influenza virus harbouring amino-acid substitutions that impair the functionality of either domain. The RBD- and ED-mutant viruses were compared to their wt- counterpart in vivo and in vitro, notably in chicken infection and avian cell culture models.

**Results:**

The double substitution R38A-K41A in the RBD dramatically reduced the pathogenicity and replication potential of the virus, whereas the substitution A149V that was considered to abrogate the IFN-antagonistic activity of the effector domain entailed much less effects. While all three viruses initiated the viral life cycle in avian cells, replication of the R38A-K41A virus was severely impaired. This defect was associated with a delayed synthesis of nucleoprotein NP and a reduced accumulation of NS1, which was found to reach a concentration of about 30 micromol.L^− 1^ in wt-infected cells at 8 h post-infection. When overexpressed in avian lung epithelial cells, both the wt-NS1 and 3841AA-NS1, but not the A149V-NS1, reduced the poly(I:C)-induced activation of the IFN-sensitive chicken Mx promoter. Unexpectedly, the R38A-K41A substitution in the recombinant RBD did not alter its in vitro affinity for a model dsRNA. When overexpressed in avian cells, both the wt- and A149V-NS1s, as well as the individually expressed wt-RBD to a lesser extent, enhanced the activity of the reconstituted viral RNA-polymerase in a minireplicon assay.

**Conclusions:**

Collectively, our data emphasized the critical importance and essential role of the RNA-binding domain in essential steps of the virus replication cycle, notably expression and translation of viral mRNAs.

## Background

Influenza A viruses (IAVs) pose a permanent threat to human and animal health, as illustrated by the 2009 H1N1 pandemic and the frequent occurrence of epizootics with highly pathogenic avian influenza viruses (e.g. H5N1 and H5N8) across the world. Their genome consists of eight single stranded RNAs of negative polarity, which collectively code for up to fifteen proteins [1–4]. Most of these genes harbour determinants that modulate the fitness, replication potential, pathogenicity and the host range of the virus, in birds and mammals [5–7].

The non-structural protein NS1 of influenza viruses is considered as the major interferon antagonist of the virus. This ~ 230-residue protein is highly expressed in the infected cell, where it exhibits several pro-viral activities [8, 9]. Its homodimeric RNA-binding domain (RBD, amino-acids 1-73) interacts with several RNAs, including viral mRNAs [10–14], while its Effector Domain (ED, amino-acids 80-202) is a non-obligate dimer that interacts with several cellular proteins. A linker region connecting the two structured domains [15, 16] confers plasticity to the quaternary structure, while the C-terminal tail (CTT, amino-acids 202-230) has the properties of an intrinsically disordered region [16].

Several studies have shown that substitutions of critical amino-acids in NS1, as well as various truncations, result in viruses with dramatically reduced pathogenicity. These notably include alanine replacement of residues within the RNA-binding domain that directly interact with RNA, and C-terminal truncations that totally or partially remove the effector domain [17–20]. In addition to those rationally designed mutations that were introduced into genetically-engineered viruses, some substitutions were demonstrated to cause the distinctive attenuated phenotype of some field-isolated avian influenza viruses [21–23]. Although most of these attenuated viruses have been characterized in depth in vivo and in vitro, to our knowledge the respective contributions of the RNA-binding- and Effector-domains to the activities of NS1 and to the pathogenicity of the virus have not been characterized.

Our objective in the present study was to elucidate the contribution of each of NS1’s two domains (i.e. RNA-binding domain and Effector domain) to its activities and to the viral phenotype. To this end, we rescued a virus harbouring a well-characterized double-substitutions in its RBD (R38A-K41A) that is known to invalidate the function of the RBD, along with a virus harbouring substitution A149V in NS1’s ED that was previously shown to abrogate the inhibitory activity of NS1 towards type I-IFN production in avian cells [22, 24]. The phenotypes of the mutant viruses were compared to that of the parental strain, in vivo after challenge of chickens, and in vitro using avian cell culture and chicken egg infection models. Our results showed that the double substitution in the RBD dramatically reduced the pathogenicity and replication potential of the virus, while the A149V substitution in the ED had only a modest effect.

## Methods

### Viruses

The low-pathogenicity avian influenza (LPAI) virus A/Turkey/Italy/977/1999 (H7N1) [25] was a kind gift of Ilaria Capua (Istituto Zooprofilattico Sperimentale Delle Venezie, Legnaro, Italy). Site-directed mutagenesis was performed on the NS segment using the QuikChange II kit (Agilent Technologies) according to the manufacturer’s protocol. The double substitution R38A-K41A was introduced using a primer pair harbouring two modified codons (codon 38 AGA > GCC and codon 41 AAG > GCG), while substitution A149V was introduced by changing codon GCA > GTG. The three recombinant IAVs (wt, 3841AA and A149V) were rescued by reverse genetics as described previously [26]. Virus stocks were prepared by one round of amplification in 10 day-old embryonated chicken eggs at a multiplicity of infection (m.o.i.) of 2 plaque-forming units (PFU) per egg. The identity of the amplified viruses was verified by sequencing their NS gene segment using reverse transcription (RT)-PCR.

### Chicken experiments

All animals were kept and treated in strict compliance with the Good Animal Practice rules as defined by the relevant national and local animal welfare authorities, and all animal experimental interventions were approved by the local ethics committee (approval number 2010/7, Comité d’Ethique pour l’Expérimentation Animale, Région Centre-Val de Loire).

Four-week-old specific-pathogen-free (SPF) histocompatible B13/B13 White Leghorn chickens were housed in biosafety level 3 cabinets under negative pressure with HEPA-filtered air. Briefly, four groups of 24 (wt), 22 (R38A-K41A), 23 (A149V) and 10 (mock) birds were inoculated intra-tracheally with 5 × 10^5^ EID_50_ (0.1 ml) of each virus, along with 10^6^ EID_50_ (0.2 ml) in the choanal cleft, while in the mock group the virus suspension was replaced by PBS. Birds were carefully monitored daily during the course of the trial (one week). Four birds at days 1, 2, 3 and 4 post-inoculation (p.i.) and 1-3 birds at day 7 p.i. for each virus-inoculated group were euthanized and necropsied, along with 2 mock-inoculated birds at each of the indicated times. Swabs and tissue samples from lung, kidney, and brain were collected from each animal and either frozen at − 80 °C until use for further downstream analyses (viral RNA quantification) or fixed in 10% neutral buffered formalin for subsequent histopathological evaluation.

### Quantification of viral RNAs

Tissue samples (50 mg) were transferred in 1 ml of PBS, and dissociated mechanically using a tissue grinder (Retsch, Germany). The QiaAmp viral RNA mini kit (Qiagen) was used to prepare viral RNAs (vRNAs) from 140 μl of tissue homogenates, according to the manufacturer’s recommendations. M-segment vRNAs were quantified through real-time RT-PCR, using the Superscript III Platinum SYBR Green one-step quantitative RT-PCR kit (Invitrogen) and a Chromo 4 thermocycler (Bio-Rad). The sequences of the primer pairs targeting the M-segment as well as the qRT-PCR conditions were previously described [27]. qRT-PCR values below the detection threshold, i.e., 200 copies, were arbitrarily replaced by 1.

### Histopathological evaluation

Formalin-fixed tissues were embedded in paraffin wax and 6 μm-thick sections were cut and routinely stained with the hematoxylin-eosin-saffron procedure. Histologic observation was then performed and each lesion recorded. For lung tissue, semi-quantitative assessment of broncho-interstitial lesions was performed and the corresponding individual score attributed as follows: 0 = no lesion; 1 = focal presence of bronchial lesions associated with moderate afflux of mononuclear cells in the surrounding parenchyma; 2 = multifocal presence of bronchial lesions with marked peripheral afflux of inflammatory cells occupying 10 to 30% of the lung parenchyma; 3 = multifocal bronchial lesions with severe infiltration of more than 30% of the parenchyma by inflammatory cells.

### Cells, infections and virus titration

MDCK and chicken lung epithelial CLEC213 cells [28] were grown in Eagle’s minimal essential medium (EMEM) and Dulbecco’s modified Eagle’s medium (DMEM-F12), respectively, supplemented with 7.5% fetal calf serum. All infections were performed with DMEM supplemented with 0.2% bovine serum albumin. TPCK-treated trypsin (Worthington, 0.4 μg/ml) was added in the case of multiple-cycle growth assays.

### Multicycle growth kinetics

Subconfluent monolayers of MDCK or CLEC213 cells were prepared in 75cm^2^-flasks. The cells were virus-infected at an m.o.i. of 0.001 PFU/cell. Following 1 h of adsorption at 37 °C, the cells were further incubated in serum-free DMEM containing 0.4 μg/ml of TPCK-treated trypsin. Supernatant samples were harvested at the indicated times post-infection and subsequently titrated by plaque assays on MDCK cells as described previously [29].

### Immunodetection of NS1

Subconfluent monolayers of CLEC213 cells in 24-well plates (grown on glass coverslips for immunofluorescence) were virus-infected at an m.o.i. of 0.5 PFU/cell. At the indicated times, cells were washed twice with PBS, fixed for 15 min in 4% paraformaldehyde and permeabilized with PBS 0.5% TritonX100. NS1 was revealed with a NS1-specific rabbit antiserum, using Alexa-conjugated secondary goat anti-rabbit antibody (1/3000 dilution). Fluorescence imaging was done using an AxioVision (Zeiss) Digital Image System. Alternatively, cells were lysed in 100 μl of Laemmli buffer and an aliquot was used for electrophoretic separation and immunoblot detection using the NS1-specific antiserum and ECL-system detection (Advansta, USA). Monoclonal antibody 9G8 (Santa Cruz Biotechnology) was used to detect NP.

### Plasmids, transfections and luciferase reporter assays

A eukaryotic expression vector encoding wt-NS1 (pCIwt-NS1) was constructed by sub-cloning the coding sequence between the *Xho*I and *Not*I sites of the pCI plasmid (Promega). In order to prevent production of spliced mRNAs, the splice-donor and splice-acceptor sites were both invalidated by point mutations [30]. Substitutions within NS1, as well as N-terminal and C-terminal truncations that were designed to express the RBD and ED were introduced using the QuikChange II kit.

For the chMX-Luc reporter assay, subconfluent monolayers of CLEC213 cells in 24-well plates were co-transfected, using the Lipofectamine 2000 transfection reagent (Life Technologies), with (i) 100 ng/well of pCI (empty vector control) or pCI-NS1 harboring the different variants of NS1, together with (ii) 100 ng/well of pGL3-chMx-Luc encoding the Firefly luciferase under the control of the chicken Mx promoter [31] (kindly provided by Dr. Nicolas Ruggli, IVI, Mittelhäusern, Switzerland), and (iii) 10 ng/well of pRL-TK (Promega) encoding the Renilla luciferase as a transfection control. Twenty-four hours post-transfection, cells were either treated with 1 μg/ml of the RIG-like receptor agonist LyoVec-poly(I:C) (L-poly(I:C)) (Invivogen), or left untreated (*n* = 3 wells for each condition). Twenty-four hours after L-poly(I:C) treatment, cells were lysed and activation of the chicken Mx-promoter was revealed by using the Dual-luciferase reporter assay system (Promega) and a GloMax-Multi microplate luminometer (Promega). For each L-poly(I:C)-treated well, the calculated foldchange is the ratio of the normalized Mx-Luc signal to the geometric mean of the normalized signals in the three untreated wells.

For the viral polymerase assays, we used the pHW-derived plasmids for the PA, PB1, PB2 and NP segments of the virus A/tk/Italy/977/1999 (H7N1) that allow the CMV promoter-driven expression of intronless mRNAs. Plasmid pgHH21-vNA-Luc [32] directs, under the control of a chicken RNA PolI promoter, the expression of a viral-like RNA containing the Firefly Luciferase ORF flanked by the extremities of the NA segment of influenza virus A/WSN/33. Subconfluent CLEC213 cells in 24-well plates (one technical triplicate for each condition) were co-transfected using the Fugene reagent (Promega) with (i) 150 ng/well of each of the pHW-PA, pHW-PB1, pHW-PB2 plasmids, (ii) 200 ng/well of the pHW-NP, pCI-NS harboring the different variants of NS1 and pgHH21-vNA-Luc plasmids, together with (iii) 5 ng/well of pRL-CMV (transfection control) encoding the Renilla luciferase under the control of the CMV promoter.

### Gel-shift assay

The RBD (aa 1-73) of A/turkey/Italy/977/1999 (H7N1), after PCR-amplification with appropriate primers, was ligated to the *Nde*I-*BamH*I digested pET15b(+) DNA (Novagen). The R38A-K41A double substitution was introduced using the QuikChange kit. Proteins were overexpressed in Rosetta2-(DE3) bacteria and purified through Nickel affinity (Histrap HP, GE Healthcare) followed by ion exchange chromatography (POROS® HS, Applied Biosystems™). After thrombin cleavage, the proteins were further separated by gel filtration (Superdex S75, GE Healthcare). Synthetic RNA shDM03 (Eurogentec) that was 5’end-labeled with ^32^P was heated (5 min. at 85 °C), then snap-cooled and incubated 30 min. on ice with increasing concentrations of protein in a 10 μl-reaction mixture (10 mM Tris-Cl, 1 mM EDTA, 0.1% bovine serum albumin, 10% glycerol, 50 mM NaCl, pH 8). RNA and RNA-protein complexes were separated as described [12]. Gels were then dried and revealed using a Typhoon-Trio laser scanner.

### Statistical analyses

**Fig. 1:**
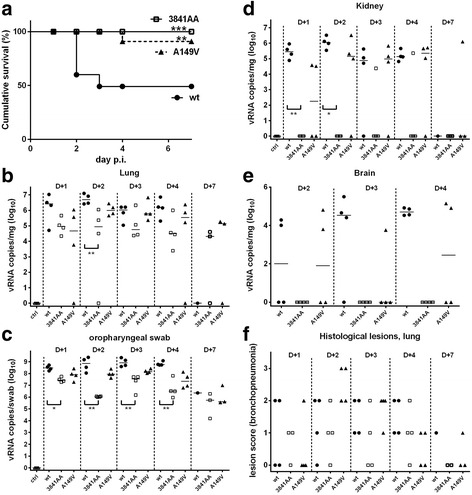
Virus pathogenicity. Four-week old SPF chickens were inoculated with the three wild-type (wt) and mutant viruses. (**a**) Deaths were recorded daily, and a survival curve was established. *** *P* < 0.001, ** *P* < 0.01 in a Mantel-Cox log-rank test comparing the considered mutant with the wt-virus. (**b-e**) At the indicated times (D + 1 to D + 7 p.i.), M-segment viral RNA was quantified in the indicated tissues or swabs, and expressed as log10 of vRNA copies per mg of tissue or per swab. Wt, filled circles; 3841AA, open squares; A149V, black triangles. Null qRT-PCR values were arbitrarily assigned a + 1 value (10^0^). ** *P* < 0.01, * *P* < 0.05 in pairwise Dunn’s post-tests comparing the considered mutant with the wt-virus in panels b, c and d. (**f**) Histological lesions of interstitial broncho-pneumonia in the lungs of the virus-infected birds were scored

**Fig. 2:**
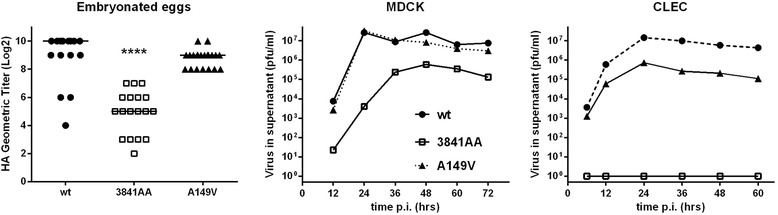
Growth properties of the three viruses. (*Left panel*) The three indicated viruses were inoculated in embryonated chicken eggs to determine virus titres (EID_50_) and the HA titres in the allantoic fluids of individual eggs were recorded. **** *P* < 0.0001 in pairwise Dunn’s post-tests comparing the considered mutant with the wt-virus. (*Middle and right panel*) The three indicated viruses were subjected to a multicycle growth assay after infection (m.o.i. = 0.001 PFU/cell) of MDCK and avian CLEC213 cells. Supernatants that were collected at the indicated times p.i. were titrated in MDCK

All statistical analyses were performed using Graphpad Prism 6.05. Kruskall-Walis non parametric tests and Dunn’s post-tests were used to analyze in vivo data (Figs. 1 and 2)**.** For the in vitro assays in transfected cells, groups of values (Figs. 5c and 7) were compared through a one-way Anova and subsequently compared pairwise with the wt- or the vector-control group by a Dunnett’s test.

## Results

### Viral replication and pathogenicity in inoculated chickens

The Low-Pathogenicity avian influenza (LPAI) virus A/Turkey/Italy/977/1999 (H7N1), a direct precursor of High Pathogenicity viruses [25, 33], expresses a NS1 protein of allele B [34, 35]. We genetically engineered three variants of this virus: the recombinant wild-type (wt) virus harboured a segment 8 identical to that of the parental isolate, the mutant virus dubbed 3841AA harboured a modified segment 8 encoding an RBD-mutated NS1 with the double substitution R38A-K41A [17, 21]; and the virus named A149V encoded an NS1 protein with a substitution in the ED that was shown to critically alter the IFN-antagonist activity of NS1 [22, 24]. We then compared the replication and pathogenic properties of the three viruses in four-week-old SPF histocompatible B13/B13 White Leghorn chickens that were inoculated intra-tracheally with 5 × 10^5^ EID_50_ of each virus, along with 10^6^ EID_50_ in the choanal cleft. Infection with the wt virus caused severe respiratory symptoms and was associated with significant mortality: of the 24 wt-inoculated animals, eight died at day 2 and two at day 3. The clinical symptoms were much less severe in the A149V group, of which only one animal died at day 4. In the 3841AA group, no clinical symptoms were observed and no deaths were recorded (Fig. 1a).

At days 1, 2, 3, 4, and 7 p.i., M-segment viral RNAs (M-vRNAs) in the tissue samples were quantified by RT-qPCR in order to evaluate viral replication and shedding [36]. All mock-inoculated chickens were negative for the presence of viral RNAs. High loads of M-vRNAs were recorded in the lungs of wt-inoculated chickens from d1 to d4 p.i. (Fig. 1b). The highest loads, close to 10^7^ copies of vRNA per mg of lung tissue, were recorded at d1 and d2. High loads in the lungs were paralleled by high amounts of M-vRNAs excreted in the upper respiratory tract (up to 10^9^ copies of M-vRNA per oro-pharyngeal swab at d2 and d3, Fig. 1b, c). The kinetics of viral replication in the lung was slightly delayed with the A149V virus, for which the highest loads were observed at days 2 and 3 p.i. At day 2 p.i., M-vRNA loads in the lungs of A149V-inoculated chickens were about tenfold lower than those of wt-inoculated chickens. In addition, virus excretion in the upper respiratory tract was reduced by about tenfold in A149V-inoculated chickens, when compared to wt-inoculated animals. M-vRNA loads in the lungs and upper respiratory tract of 3841AA-inoculated animals were dramatically reduced, by ten to 100-fold relative to wt-inoculated birds. The wt-virus, and to a slightly lesser extent the A149V-virus, were found in the kidneys and brains, indicating limited systemic dissemination (Fig. 1d, e), while in the 3841AA-group, only two kidneys, and no brain tissues, were found vRNA-positive.

Lung lesions were observed only in virus-inoculated chickens. Macroscopic lesions were mainly observed at d2 and d3 in wt- and A149V-inoculated chickens, and were not recorded in 3841AA-inoculated birds. Histological examination of the lungs of wt- and A149V-inoculated chickens revealed severe lesions of peribronchiolar interstitial pneumonia, associated with intraluminal presence of a fibrinous exudate and some degenerating heterophils, typical of exudative bronchitis and parabronchitis (Fig. 1f). This exudate obstructed airways in severe cases. No lesions were observed in the other examined tissues. In spite of the symptomless infection, mild histological lesions were observed in 3841AA-inoculated animals, consisting essentially of foci of infiltrating mononuclear inflammatory cells scattered in the lung parenchyma around airways.

### NS1 mutants replicate to lower levels, both in embryonated eggs and in cultured cells

We reasoned that the distinct replication efficiencies and pathogenicities of the three viruses could be paralleled by similar properties in embryonated chicken eggs or in infected cells. In embryonated eggs, the A149V virus grew to HA titers that were slightly lower than those of the wt virus, while the 3841AA-mutant grew to dramatically reduced levels, about 5 log_2_ units lower than those of the wt virus (Fig. 2).

In multicycle growth assays in MDCK cells, the wt- and A149V-viruses showed virtually identical replication kinetics reaching 10^7^ PFU/ml in the supernatant as early as 24 h post infection, while the growth of the 3841AA-virus was clearly delayed, with a maximum titer that remained at least tenfold lower than those of the two other viruses. In CLEC213 cells, a chicken lung epithelial cell line that we have previously described [28], the growth of the A149V virus was reduced by about tenfold as compared to its wt-counterpart, while no virus was found in the supernatant of the 3841AA-inoculated cells, in spite of repeated attempts.

### Mutations alter the accumulation and subcellular distribution of NS1 in infected cells

**Fig. 3:**
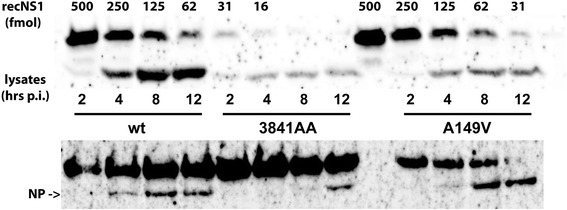
Accumulation of NS1 in virus-infected CLEC213 cells. Subconfluent CLEC213 cells in a 24-well plate were infected (0.5 PFU/cell) and lysed at the indicated times post-infection. Proteins in the lysates (0.8% of the 100 μl-lysate) were separated through SDS-PAGE, then transferred to a nylon membrane. For NS1 quantification, increasing amounts of recombinant NS1 protein (16 to 500 femtomoles) were loaded on the same gel 20 min after the lysates. Except for a C-terminal (His)_6_ extension, the recNS1 is identical to the viral NS1; its apparently slower migration profile only results from the delayed loading. NS1 was revealed through immunoblot, using a polyclonal rabbit serum. Lower panel: the same lysates were used for the western-blot detection of NP (the upper band corresponds to an unidentified cellular protein that was also detected in non-infected cells)

To better understand by which mechanisms substitutions in NS1 might hamper the viral replication cycle, we first measured the accumulation of NS1 in cells infected with the three viruses. Avian CLEC213 cells were infected with the three viruses at an m.o.i. of 0.5 PFU per cell and the accumulation of NS1 in the infected cells during the course of the viral cycle was monitored by a Western-blot assay (Fig. 3). On the same gel we loaded increasing calibrated amounts of recombinant NS1 from the same virus, thus allowing us to estimate the amount of NS1 in the lysates and its cellular concentration, assuming that confluent cells in the well of a 24-well plate represent a total intracellular volume of about one microliter. From several independent experiments, we estimated that, as early as 2 h p.i., NS1 reaches a concentration of 2 to 5 micromol.L^− 1^ in wt-infected cells. At later time-points, NS1 concentration continues to increase, up to about 30 micromol.L^− 1^, a surprisingly high concentration that is of the same order of magnitude as those of the most abundant cellular proteins, such as actin [37, 38]. As shown in Fig. 3, NS1 expression was markedly reduced (by about 4 to 8-fold) in 3841AA-infected cells and slightly reduced in A149V-infected cells, compared to the levels observed in wt-infected cells. Of note, if one assumes that NS1 is homogeneously distributed in the infected cell, it is expected to passively diffuse into the budding virion: assuming a cytoplasmic NS1 concentration of 30 micromol.L^− 1^, a virion of 100 nm in diameter (~ 5 × 10^− 19^ l in volume) could contain about nine molecules of NS1, which is consistent with the published estimates [39]. Using the same infected cell-lysates, we observed that the accumulation of the viral nucleoprotein NP was considerably delayed in 3841AA-infected cells, where it was detected only at 12 h p.i., while it was detected as early as 4 h p.i. in wt-infected cells (Fig. 3, lower panel).

**Fig. 4:**
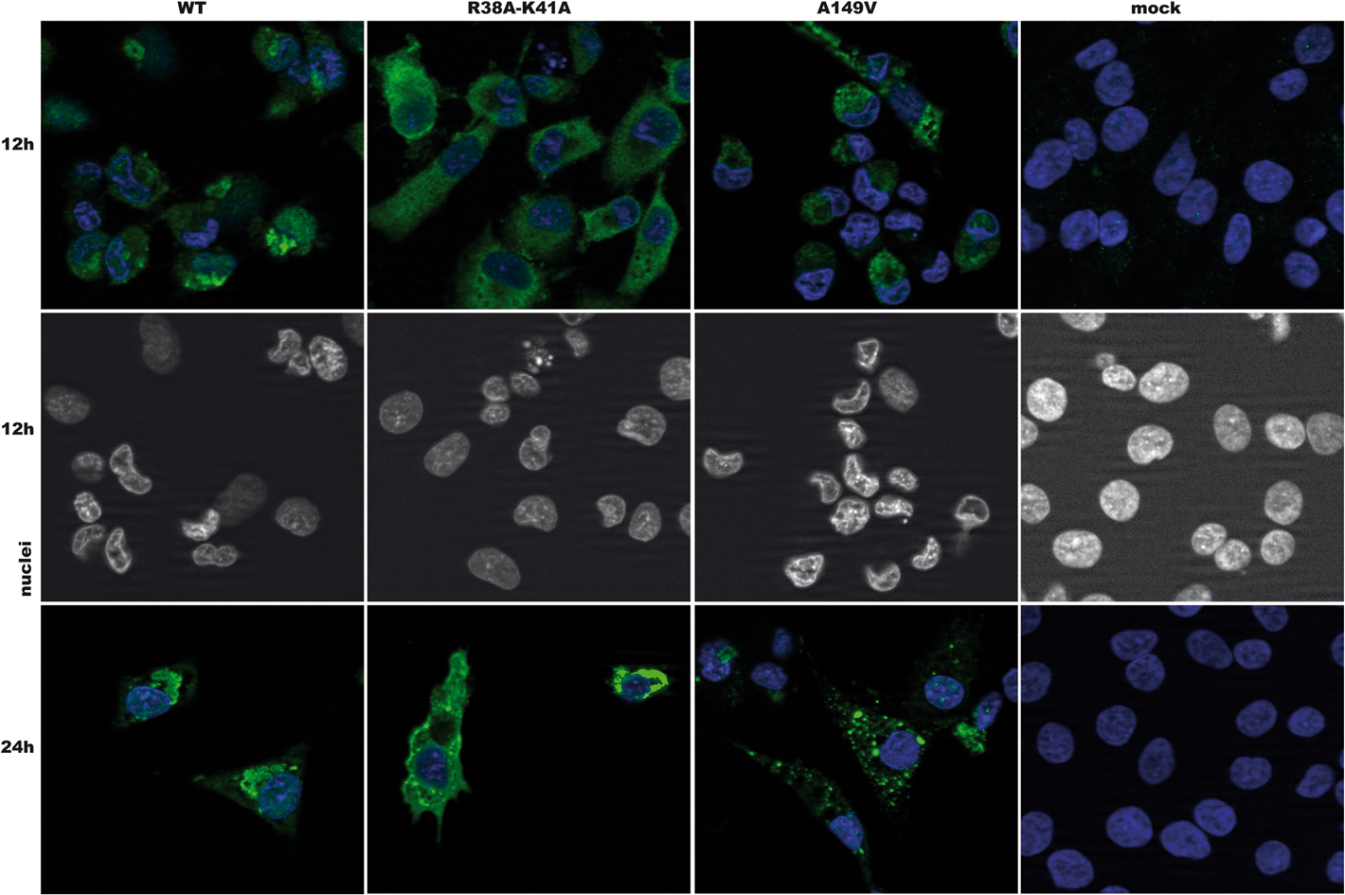
Subcellular distribution of NS1 in virus-infected CLEC213 cells. CLEC213 cells were mock-infected or infected with the indicated viruses (0.5 PFU/cell), then fixed and immunostained with an NS1-specific rabbit antiserum at 12 h or 24 h p.i. Images in the top and middle panels are from the same selected fields (middle panel shows the DAPI-stained nuclei at 12 h p.i.)

The distinct phenotypes of the three viruses likely resulted from several distinct biological properties of the NS1 variants, including their subcellular distribution which we analyzed in infected cells. Avian CLEC213 cells were infected with the three viruses at an m.o.i. of 0.5 PFU/cell, then fixed and immunostained with a polyclonal anti-NS1 rabbit serum at 12 and 24 h p.i. (Fig. 4). The three NS1 variants exhibited distinct patterns of subcellular distribution. The wt-NS1 formed crystal-like cytoplasmic inclusions, similar to those previously described for some avian influenza viruses [40]. In stark contrast, 3841AA showed an intense staining that filled the whole cytoplasm. A149V produced cytoplasmic aggregates that did not exhibit the crystal-like structures observed with the wt-NS1. All three viruses produced a cytopathic effect, as judged by the low number of adherent cells at 24 h p.i.. Furthermore, at 12 h p.i. we observed that most cells infected with the wt and A149V viruses exhibited a disfigured shape of nuclei, a feature that was much less pronounced with the 3841AA-virus.

### The three NS1 variants moderately impact the type I IFN response in avian CLEC213 cells

**Fig. 5:**
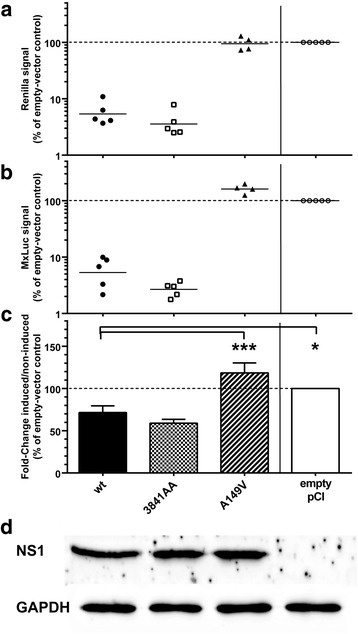
NS1 variants differentially modulate type I IFN response. CLEC213 cells in 24-well plates were transfected with the two plasmids used for the dual-luciferase assay for IFN-induced chicken Mx promoter activity, along with recombinant expression vectors encoding the indicated NS1-variants. Cells were stimulated at 24 h post-transfection by a L-poly(IC) treatment, and subsequently lysed 24 h later (48 h post-transfection). The chMx promoter-driven Firefly activity (FFL) was measured in the lysates (**b**) and normalized relative to the Tk-promoter driven Renilla-luciferase activity (**a**). (**c**) Fold-change ratios (induced/non-induced) are expressed relative to that calculated from the respective empty-vector control value set at 100%; * *P* < 0.05; *** *P* < 0.001 in a Dunnett’s test comparing each set to the wt-NS1 condition. (**d**) Western-blot detection of NS1 in transfected cells. Proteins in the lysates (1/8th of the 100 μl-lysate) were separated through SDS-PAGE, then transferred to a nylon membrane and revealed as in Fig. 3. Data are from five independent experiments, each point in (**a**) and (**b**) representing the geometric mean of a technical triplicate

We then sought to determine whether the variants of NS1 might differentially regulate the antiviral interferon response. We surmised that the antiviral response in virus-infected cells could be biased by the differing replication efficiencies of the wild-type and NS-mutant viruses. Hence we opted to measure the LyoVec-poly(I:C)-induced antiviral response in cells that transiently expressed the three NS1 variants [31]. CLEC213 cells were transfected with plasmid pGL3-chMx-Luc encoding the firefly luciferase under the control of the IFN-inducible chicken Mx promoter, along with a “normalizer” Renilla Luciferase-encoding plasmid as well as expression plasmids encoding wt- or substituted NS1s, or the empty plasmid pCI as a control. The three variants of NS1 were expressed to similar levels, as verified by a western-blot assay using a rabbit anti-NS1 polyclonal serum (Fig. 5d). Transfected cells were stimulated by treatment with the MDA5-agonist Lyovec-poly(I:C) and the onset of an IFN-I response was measured 24 h after treatment by a dual-luciferase assay. Compared to the corresponding untreated cells, the L-poly(I:C) treatment of empty vector-transfected cells induced a strong activation of the Mx promoter, that varied from 10 to 28-fold depending on the experiment. Therefore we arbitrarily set a 100% value for the foldchange in empty pCI-transfected cells in order to combine all data from five independent experiments (Fig. 5c). Transient expression of wt-NS1 diminished the L-poly(I:C)-induced Mx-promoter activation, while A149V-NS1 slightly increased the activation. Unexpectedly, the 3841AA mutant reduced the activation by about 50% relative to empty-vector transfected cells. Interestingly, in these experiments we consistently observed a dramatic decrease in the activity of the two types of luciferase in cells expressing NS1 with an intact effector domain (Fig. 5a, b): in wt-NS1-transfected cells the activities of both Firefly and Renilla luciferases were 6-8% of those recorded in empty vector-transfected cells, while in cells transfected with the 3841AA variant they were further reduced to 2-3%. Substitution A149V in the effector domain completely abolished this effect.

### Mutated RBD still binds double-stranded RNAs

**Fig. 6:**
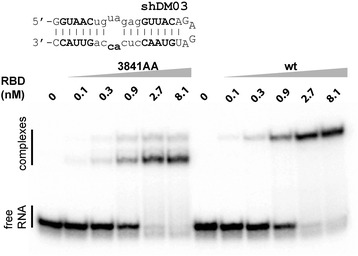
In vitro interaction of wt- and mutated RBDs with model dsRNA (electrophoretic mobility shift assay). ^32^P-labeled, synthetic RNA shDM03 (depicted above as MFold-predicted structure) at a final concentration of 0.1 nMol.L^− 1^ was incubated with increasing concentrations (as indicated) of the dimeric wt-RBD (right panel) or mutated-RBD (left panel). RNA-protein complexes were electrophoretically separated in a 10% non-denaturing polyacrylamide gel, and were subsequently revealed using a Typhoon-Trio laser scanner

It is generally considered that the attenuated phenotype of viruses harboring the double substitution R38A-K41A results from the fact that the mutated RBD has lost its ability to bind double-stranded RNAs [17, 41]. In order to confirm this issue, we compared the in vitro RNA-binding properties of the wt- and mutated RBDs. Both RBDs were purified from overexpressing bacteria, and used in a gel-shift experiment using a model double-stranded RNA that we had designed previously [12]. As shown in Fig. 6, the wt-RBD strongly bound the model dsRNA with an apparent K_D_ close to 1 nM. Unexpectedly, the double substitution did not substantially alter the affinity of the RBD for this model dsRNA, as shown by the virtually unchanged K_D_. However, the distinct migration profiles observed with the two variant RBDs suggested that the RBD-RNA complexes were structurally different.

### NS1 modulates the activity of the viral polymerase

**Fig. 7:**
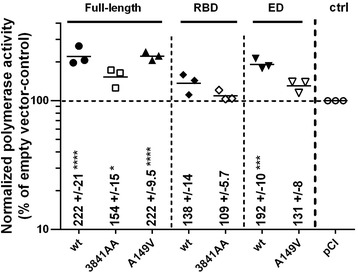
Complementation of the viral polymerase activity by NS1. CLEC213 cells in 24-well plates were transfected with the plasmid set of the pHW-based H7-minireplicon system (see Methods section), along with an NS1-expression vector or an empty vector control. Twenty-four hours later, cells were lyzed and the Firefly-luciferase activity of the lysates, resulting from translation of the polymerase-transcribed mRNA, was measured and normalized relative to the Renilla-luciferase through a dual luciferase assay. Each point represents the normalized polymerase activity (geometric mean of a technical triplicate) relative to the empty-vector control in a given experiment (numbers are the mean and s.e.m from three independent experiments). The “no-PB1” control values (not shown) were less than 0.1%. * *P* < 0.05; *** *P* < 0.001; ****P < 0.0001 in a Dunnett’s test comparing each set to the control (empty pCI) condition

Both the in vitro and in vivo data pointed to a dramatically reduced replicative efficiency of the 3841AA-virus, while the replication fitness of the A149V virus appeared to be moderately affected. This suggested that NS1, and notably its RBD, might modulate the activity of the viral polymerase complex. To address this issue we set up a minireplicon assay: CLEC213 cells were transfected with the four pHW2000-derived expression vectors allowing the reconstitution of the viral polymerase complex (PA, PB1, PB2, NP), along with a Firefly-luciferase-encoding minireplicon and NS1 expression vector. The Firefly plasmid reports the expression of a viral RNA mimic in which the Luciferase ORF is flanked by the extremities of the NA segment of influenza virus A/WSN/33. Transcription of this vRNA mimic by the viral polymerase produces an artificial viral mRNA encoding the Firefly-luciferase, whose activity was measured by a dual luciferase assay. We observed that the expression of the distinct variants of NS1 (truncations or substitutions) increased the apparent polymerase activity to various degrees (Fig. 7): (i) the wt-ED induced a ~twofold increase of the apparent polymerase activity, while the wt-RBD had a lesser impact (~ 38% increase); (ii) substitutions in either individually expressed domain (3841AA-RBD or A149V-ED) reduced or nearly abolished the positive effect observed with the wt-domain; (iii) the full-length NS1 induced a twofold increase of polymerase activity; (iv) the double substitution in the RBD, but not the A149V substitution in the ED, reduced the effect of the full-length protein. The latter observation could result from the fact that the effect of the A149V substitution was compensated for by the presence of the wt-RBD. Similarly to what we observed in the Mx-Luc assay, all three conditions where cells expressed a wt-ED (wt and 3841AA full-length NS1, wt-ED) resulted in a ~twofold reduction in the activity of the normalizer Renilla Luciferase. This probably resulted from a negative effect of the wt-ED on the expression of cellular mRNAs, and could also partly bias our data and their interpretation (as discussed below).

## Discussion

In an attempt to delineate the respective roles of the two structured domains of NS1, we genetically engineered three variants of the Low Pathogenicity Avian influenza virus A/tk/Italy/977/99(H7N1). On the one hand, we introduced in the RNA-binding domain the double substitution R38A-K41A, which is considered to abrogate its RNA-binding properties [17, 20, 41]; on the other hand, the ED was modified by substitution A149V, which was previously shown to alter the IFN-antagonist activity of NS1 in avian cells [22, 24]. In a well-established chicken model of avian influenza virus infection [42, 43], the wt-virus induced severe respiratory symptoms and deaths while the symptoms were much less severe in the A149V-inoculated birds and infection remained silent in the 3841AA-inoculated birds. The different pathological outcomes were reflected both by the M-vRNA loads in tissues and in the histopathological findings, while the distinct replication efficiencies of the three viruses in vivo were correlated to similar phenotypes in vitro: replication of the A149V mutant was slightly reduced relative to that of the wt-virus in both embryonated eggs and in the chicken lung epithelial cell line CLEC213, while that of the 3841AA-virus was severely reduced in eggs and non-measurable in the avian cell line.

In these cells, all three viruses initiated a replication cycle, as evidenced by the accumulation of viral protein NS1, and all three induced a cytopathic effect (Fig. 4). Nevertheless, the 3841AA virus seemed unable to produce infectious viral progeny in CLEC213 cells, as demonstrated by the multi-cycle growth assay. At least three mechanisms could explain such a defect: (i) a reduced replication rate, either inherent to the virus or resulting from an increased antiviral defense, (ii) a reduced synthesis of viral proteins, or (iii) a defect in the late steps of the viral cycle (assembly and release). Reduced IFN-antagonistic activities of NS1 may account for an increased antiviral response in cells infected with the two NS-mutant viruses. Indeed we observed that the transiently-expressed wt-NS1 reduced the L-poly(I:C)-induced antiviral response. Substitution A149V in the ED abolished this effect, in agreement with the critical role of the effector domain in dampening the IFN response [22, 24, 44]. However, the R38A-K41A substitution did not abrogate this effect; on the contrary, it further reduced the activity of the IFN-responsive Mx promoter. This observation contrasts with the findings from previous studies with mutant influenza A viruses harbouring the same amino-acid substitutions in NS1’s RBD. Specifically, transient expression of wt-NS1 from the WSN virus was shown to prevent the Sendai virus-induced IFN-beta response in 293 T cells, while WSN-NS1 harbouring the double substitution R38A-K41A was unable to reduce this IFN-beta response [17]. The discrepancy between our data and the literature could originate from at least four distinct experimental parameters: (i) NS1 allele and proteotype; (ii) the cell-type and their species origin; (iii) the interferon-induction treatment; and (iv) the assay used to evaluate the interferon response. Firstly, distinct proteotypes of NS1, both within and between its two alleles, can differ by their biological activities [34]; while the WSN virus harbors allele A of NS1, our study focused on allele B NS1 from the avian H7N1 virus. Secondly, while we investigated, in avian lung epithelial cells, the L-poly(I:C)-induced activation of the MDA5 pathway by measuring the activity of the IFN-induced chicken Mx gene-promoter, Donelan et al. [17] evaluated the IFN-response at a broader level by measuring, in Sendai virus-treated mammalian 293 T cells, the activity of the IFN-beta promoter. In addition, we speculate that the R38A-K41A NS1 may still bind the synthetic dsRNA of L-poly(I:C) and thereby prevent the activation of the RIG-like receptor pathway. On the one hand, this could be facilitated by the diffuse cytoplasmic distribution of this mutant NS1 that we observed in infected cells. On the other hand, we observed that the double substitution R38A-K41A did not substantially alter the binding of the recombinant RBD to a model dsRNA. Furthermore, and contrary to the common belief, this double substitution likely does not totally invalidate the RBD. Indeed Schierhorn et al. recently showed that NS1’s RBD was endowed with at least two distinct activities that each critically involved partially overlapping sets of amino acids. While R38, like R35 and R46, is critical for binding of the PR/8 virus-derived NS1 to poly-IC, R35 and R46, unlike R38, also have a critical role in binding to PKR and preventing its phosphorylation [45]. Our data and this discrepancy clearly highlight the need for a better understanding of the multiple pathways of the interferon/antiviral response in avian cells, including their modulation by NS1 as well as comparisons with the interferon response in mammalian cells.

Our data showed that the three NS1 variants differentially modulated the activity of the viral polymerase. Using a minireplicon assay in transfected cells, we showed that NS1 by itself increased by nearly twofold the activity of the transiently-expressed viral polymerase. The polymerase enhancing property of NS1 was reduced by the double substitution R38A-K41A, and virtually unaltered by the A149V substitution. Although each individually expressed domain also increased the apparent polymerase activity, we observed that the expression of a protein harbouring a wt-ED (wt-ED, wt-NS1 and 3841AA-NS1) consistently reduced to ~ 50% the activity of the normalizer Renilla Luciferase. As a result, the twofold increase of the normalized polymerase activity that we observed in these three cases could result in a large part from this bias. Therefore, if we disregard these potentially biased observations, our data indicated that (i) the individually-expressed wt-RBD, but not its 3841AA mutant, induced a ~ 38% increase in the polymerase activity; (ii) the individually-expressed A149V-ED induced a ~ 30% increase in the polymerase activity; (iii) when adjoined (i.e. in A149V-NS1), these two domains acted synergistically, resulting in a more than twofold increase in the polymerase activity. In short, the wt-RBD enhanced the activity of the viral polymerase, an effect that was further increased when it was linked to the ED. This is in line with several independent studies showing that NS1 interacts with the viral polymerase and can regulate its activity [46–48]. More specifically, this interaction was shown to rely on the binding of NS1’s RBD to the viral nucleoprotein NP, a binding that was abolished by the double substitution R38A-K41A [49]. If NS1 directly enhances the activity of the viral polymerase through this interaction, it may also act indirectly through interacting with host factors such as DDX21 and hnRNP-U that were shown to modulate the polymerase activity [50, 51].

The inability of the 3841AA virus to produce infectious viral progeny in the avian cells likely also resulted from a reduced synthesis of viral proteins. Indeed, while NS1 reached a very high concentration in wt virus-infected cells, its concentration in 3841AA-infected cells was reduced by about 4 to 8-fold relative to that in wt-infected cells, and slightly reduced in A149V-infected cells. We also observed a severely reduced yield of the viral nucleoprotein NP in the 3841AA-infected cells (Fig. 3), which likely was indicative of a reduced or delayed synthesis of all viral proteins. Indeed NS1 was shown to specifically enhance the translation of viral mRNAs [13, 52] notably through binding of its N-terminal region to the strictly conserved sequence motif at the 5’end of viral mRNAs and increasing their translation-initiation rate [13, 53]. We hypothesize that the 3841AA NS1 no longer recognizes the viral-specific motif [12], and as a consequence has lost its translation-enhancing activity.

In addition to the defects discussed above, other mechanisms that we have not investigated at the late steps of the viral cycle may also account for the dramatically attenuated phenotype of the 3841AA mutant virus. First, it was recently shown that NS1, unlike its R38A-K41A mutant, binds both NXF1 and the late viral mRNAs (notably HA-mRNA and the unspliced viral M1 mRNA), thereby directing the latter to the nuclear export pathway [54]. The R38A-K41A NS1 mutant of the PR/8 virus was shown to exhibit a severe defect in the nuclear export of HA- and M1-mRNAs. Independently from its requirement in the expression of the late viral genes, NS1 could also be involved in some steps of the virion formation: an in-depth analysis of a NS1-mutant virus identified a block in the production of virus particles, pointing to a possible involvement of NS1 in viral assembly [55]. Incidentally, the 3841AA virus has the properties required for a live-attenuated vaccine candidate [56].

As for the ED, our data show that it is involved in two activities that are both abolished by the A149V substitution. Firstly, in agreement with previous reports [22, 24], we observed that wt-NS1, unlike its A149V variant, decreased the IFN-response of L-poly(I:C)-stimulated avian cells, as measured by the activity of the chMx-promoter. Secondly, the expression of either wt-ED or full-length NS1 with an intact ED consistently reduced the expression of the luciferase mRNA that we used to normalize our luciferase-based assays. Compared to that measured in empty-vector control-cells, the activity of the normalizer Renilla luciferase in all the conditions where cells expressed a wild-type ED was reduced to ~ 50% at 24 h post-transfection (minireplicon assay) and to a few percent at 48 h post-transfection (Mx-Luc assay). Substitution A149V consistently abolished this effect. This emphasizes the major role of the effector domain in inhibiting the maturation and nuclear export of non-viral mRNAs, relying on its interaction with Cleavage and Polyadenylation Specificity Factor [57, 58]. Given that alanine 149 is deeply buried within the hydrophobic core of the ED [59], we surmise that its replacement with the bulky valine residue destabilizes the structure of the effector domain [22].

## Conclusions

Our data clearly showed that the double substitution R38A-K41A severely impacted the viral phenotype, much more than the A149V substitution. This severe attenuation stems from at least three, and possibly four combined major defects: (i) a lower activity of the viral polymerase, which is no longer enhanced by the RBD-impaired NS1 (present data); (ii) a reduced translation of viral mRNAs (present data); (iii) a blocked nuclear export of late viral mRNAs [54]; (iv) possibly an impaired virion assembly [55]. The severely altered phenotype of the 3841AA-mutant, as compared to the slighter impact of the A149V substitution, emphasized the essential role of the RBD and the relative dispensability of the effector domain, which have been discussed previously [13] and are further supported by the unaltered pathogenicity of a modified bat-influenza virus expressing an RBD-only NS1 [60]. Altogether, our data and the published literature suggest that NS1 acts much more at the core of the viral cycle, through interactions of its RBD with virus-derived RNAs and proteins, than at its periphery through its ED being involved in the interactions with the host cell.
